# Protocol of a study to benchmark occupational health and safety in Japan: W2S-Ohpm study

**DOI:** 10.3389/fpubh.2023.1191882

**Published:** 2023-11-03

**Authors:** Tomohisa Nagata, Kiminori Odagami, Masako Nagata, Nuri Purwito Adi, Koji Mori

**Affiliations:** ^1^Department of Occupational Health Practice and Management, Institute of Industrial Ecological Sciences, University of Occupational and Environmental Health, Japan, Kitakyushu, Japan; ^2^Department of Occupational Medicine, School of Medicine, University of Occupational and Environmental Health, Japan, Kitakyushu, Japan; ^3^Department of Community Medicine, Faculty of Medicine, Universitas Indonesia, Jakarta, Indonesia

**Keywords:** protocol, occupational health and safety, benchmarking, web survey, sampling method

## Abstract

We aim to conduct a prospective cohort study to benchmark occupational health and safety in Japan. Here, we describe the detailed protocol for the baseline survey based on the Checklist for Reporting Results of Internet E-Surveys. We conducted the baseline survey for the prospective cohort study in 2022. Our target population was workers in Japan aged 20 years or older, who we sampled to be representative of the Japanese workforce, stratified by sex, age, and region. Among 59,272 registered monitors who answered the initial screening questions, 29,997 completed the survey. After excluding 2,304 invalid responses, we used 27,693 valid participants in our final analysis. The number and mean age of men were 15,201 (55%) and 46 years; those of women were 12,492 (45%) and 45 years. With respect to sex, age, and regional composition, our sampling was representative of Japan’s working population. Our sampling for employment status and industry yielded almost the same proportions as a government-led representative sampling of workers in Japan.

## Introduction

Occupational health and safety should be managed continuously by improvements based on the goals and plans of management systems at both the company and national levels. The International Labour Organization (ILO) Convention No. 187 was established in 2006 (1). Its Article 5 states as follows: “Each member shall formulate, implement, monitor, evaluate and periodically review a national program on occupational safety and health in consultation with the most representative organizations of employers and workers.” As the first country to adopt that convention as a standard, Japan has operated the Occupational Safety and Health Program since 1958 (2). That program is in the form of a 5-year plan, and it is reviewed in accordance with ongoing developments. Such reviews demand highly accurate statistical details of occupational health and safety activities as well as occupational accidents.

Japan’s government regularly obtains information about occupational health and safety. Investigations into occupational safety and health in that country have been conducted as general statistical surveys under the Statistics Law (3). To determine the current situation and ensure nationwide representativeness, we adopted a stratified sampling method. Those government surveys have the advantage of large sample size and random countrywide sampling; however, there are drawbacks, such as the enormous survey costs and the fact that many are cross-sectional—not longitudinal—studies. A cross-sectional study makes it difficult to determine causality because time-series relationships cannot be included in the analysis.

When implementing activities based on an occupational health and safety management system in a company, it is necessary to clarify issues related to occupational health and safety and to set targets for activities. For this purpose, we need benchmark data on occupational accidents and employees’ health that can be compared to the company’s own situation. Since these data vary by industry, job category, company size, etc. for occupational accidents, and by sex, age, etc. for health, we need benchmark data sorted by each category. While it is true that these benchmark data are not entirely public, they are not yet available in a form that makes it easy for companies to use them for goal-setting purposes.

Online surveys are becoming more widely used in research. Many concerns have been raised about the quality of Web-based surveys (4). However, Internet surveys have the advantage of a large sample size at relatively low cost compared with paper-based surveys. Studies using online panels have been widely applied because they are inexpensive and allow for data acquisition over a short period of time (5). Surveys using online panels can be applied to provide follow-up information, thereby permitting longitudinal studies, such as prospective cohorts. In online panel surveys, sampling methods are important, and respondent bias has to be assessed.

We conducted a prospective cohort study on occupational health and safety in Japan. This paper describes the detailed protocol for that study. We report on the research protocol using the Checklist for Reporting Results of Internet E-Surveys (CHERRIES), which is a means for improving the quality of Web surveys (6).

## Materials and analysis

This is a prospective cohort study conducted online. We started the baseline survey on February 28, 2022, and conducted it up to two weeks before the required sample was collected. As a result, the samples were collected by March 3, 2022. We then planned to conduct a follow-up survey once a year. The target population for the survey was workers in Japan aged 20 years or older. The study was approved by the Ethics Committee of the University of Occupational and Environmental Health, Japan (R3-076).

### Sampling plan

To ensure that the target population was representative of workers in Japan, the participants were sampled so that the proportion of workers stratified by sex, age, and region was the same as the actual Japanese workforce (7). We categorized as follows: sex into two groups (men, women); age into eight groups (20–29, 30–39, 40–44, 45–49, 50–54, 55–59, 60–64, and ≥ 65 years); and region into 10 groups listed from north to south (Hokkaido, Tohoku, South Kanto, North Kanto and Koshinetsu, Hokuriku, Tokai, Kinki, Chugoku, Shikoku, and Kyushu and Okinawa). We set the target sample size at around 30,000 participants, based on a previous study (8). We assigned the number of workers in each of the 160 collection units stratified by sex, age and region, and the sample size for each collection unit as indicated in Table 1.

**Table 1:** Participants stratified by sex, age, and region in the sampling plan.

		Age categories								
Region (prefecture)		Total (29,997)	20–29 (4,982)	30–39 (5,657)	40–44 (3,434)	45–49 (3,993)	50–54 (3,460)	55–59 (2,968)	60–64 (2,340)	≥65 (3,163)
Hokkaido (Hokkaido)	Men	633	92	112	72	82	66	66	61	82
	Women	541	82	97	61	72	66	61	41	61
Tohoku*	Men	1,079	148	199	123	133	118	118	107	133
	Women	895	128	169	107	118	102	102	82	87
South Kanto†	Men	5,249	884	1,053	603	690	618	496	363	542
	Women	4,140	828	792	460	557	496	378	261	368
North Kanto and Koshinetsu‡	Men	1,242	184	230	143	164	143	128	107	143
	Women	988	143	174	118	138	118	102	82	113
Hokuriku§	Men	670	97	123	77	87	72	66	61	87
	Women	565	87	102	66	82	66	61	46	55
Tokai||	Men	2,064	337	399	235	276	235	199	158	225
	Women	1,605	281	291	179	225	189	153	123	164
Kinki¶	Men	2,538	419	470	281	337	296	250	199	286
	Women	2,152	399	383	245	307	261	215	153	189
Chugoku**	Men	905	143	174	102	118	97	87	77	107
	Women	762	118	138	92	107	87	77	61	82
Shikoku††	Men	424	61	77	51	56	46	46	36	51
	Women	378	51	66	46	51	46	41	36	41
Kyushu and Okinawa‡‡	Men	1,655	255	322	194	199	169	164	158	194
	Women	1,512	245	286	179	194	169	158	128	153

The names of the prefectures in each region are shown in order from North to South.

* Aomori, Iwate, Miyagi, Akita, Yamagata, Fukushima.

† Saitama, Chiba, Tokyo, Kanagawa.

‡ Ibaraki, Tochigi, Gunma, Yamanashi, Nagano, Niigata.

§ Toyama, Ishikawa, Fukui.

|| Gifu, Shizuoka, Aichi, Mie.

¶ Shiga, Kyoto, Osaka, Hyogo, Nara, Wakayama.

** Tottori, Shimane, Okayama, Hiroshima, Yamaguchi.

†† Tokushima, Kagawa, Ehime, Kochi.

‡‡ Fukuoka, Saga, Nagasaki, Kumamoto, Oita, Miyazaki, Kagoshima, Okinawa.

### Recruitment procedure

The survey was commissioned by Rakuten Insight, Inc. (Tokyo, Japan), which has 2.2 million registered monitors. Only individuals registered with Rakuten Insight could respond to the Internet survey. Owing to the company’s information confidentiality, we were unaware of the number of initial invitations to participate; however, 59,272 registered monitors responded to the initial screening questions and participated in the survey. Those 29,997 matched the survey’s criteria (worker status, sex, age, and region). As an incentive, survey respondents were able to earn redeemable points from the company; however, again owing to the company’s information confidentiality, we did not know the number of points.

### Data retrieval

Before submitting the questionnaire, we undertook consistency or completeness checks. For inconsistency in a question, respondents were asked to view on-screen indications that their answers were inconsistent; specifically, that comprised individuals who provided meaningless characters and symbols in the free-answer column (e.g., “AAA” and “$&¥”). This approach has been shown to be effective in detecting fraudulent responses at an early point in surveys (9). We also excluded participants who responded in an excessively short period of time: we regarded such responses as invalid. As *post hoc* means to eliminate fraudulent responses, we set the exclusion criteria as follows: extremely high body weight (>300 kg); excessive height (>250 cm); and clearly incorrect answers (respondents who indicated that they were engaged in work for 0 days and 0 h; those who stated they worked over 150 h a week; those who did over 100 h overtime a week; and those who claimed they lived with 18 or more family members).

### Measurements

The survey items included basic sociodemographic characteristics, such as sex, gender, nationality, marriage status, income, employment status, and industry category. “Sex” signified biological sex and was determined as the sex on the family register at birth and birth certificate, whereas “gender” was a self-identified term. To identify “gender,” we asked the question, “Regardless of your sex at birth, please indicate the gender that most closely matches your current perceived image”; respondents chose from three options (male, female, and other). Marital status was classified into three categories: married; unmarried; and divorced or bereaved. We determined household income (total income of all cohabiting family members) over 1 year (2021), with options given in units of 1 million Japanese Yen. We classified household income into the following six categories: <4 million; 4 million–6 million; 6 million–8 million; 8 million–10 million; 10 million–12 million; and > 12 million Japanese Yen. Respondents were asked to select their employment status from the following eight options: self-employed; company executive; full-time employee; part-time work; dispatched employee; contract employee; freelance; and others. As noted earlier, we excluded non-working individuals from this study at the screening stage.

For comparison with various surveys conducted by the Japanese government, we classified industries into the following 20 categories based on the Japan Standard Industrial Classification (10): agriculture and forestry; fisheries; mining and quarrying of stone and gravel; construction; manufacturing; electricity, gas, heat supply and water; information and communications; transport and postal services; wholesale and retail trade; finance and insurance; real estate and goods rental and leasing; scientific research, professional and technical services; accommodations, eating and drinking services; living-related and personal services and amusement services; education and learning support; medical, health care and welfare; compound services; services; public sector; and unlabeled. This classification is roughly—though not perfectly—consistent with the International Standard Industrial Classification (11); thus, we believed it would allow international comparisons to be made.

We examined negative health conditions through psychological distress using Kessler 6 (K6) as well as diseases and their treatment status. K6 was developed to screen for psychological distress (12, 13), and the Japanese version has been validated (14, 15). Based on the previous studies, we applied a K6 score of 5 or higher as the cutoff for mild psychological distress and one of 13 or higher as the cutoff for severe psychological distress. Positive health conditions were examined by work engagement using a nine-item Japanese version of the Utrecht Work Engagement Scale (UWES-9) (16, 17). The total score was obtained by averaging the individual item scores (possible range, 0–6). The internal reliability and validity of the Japanese UWES-9 are acceptable (17). In addition to these, various other factors and outcomes were investigated in this survey. The types and questionnaires are listed in Table 2. We will publish these detail benchmark data in the near future.

**Table 2:** Lists of contents of the questionnaire.

Contents	Questionnaire / questions
*Personal and workplace resources*	
Job demands	The Brief Job Stress Questionnaire
Job control	The Brief Job Stress Questionnaire
Social support	The Brief Job Stress Questionnaire
Workplace social capital	Workplace social capital (WSC) in Finnish Public Sector Research
Health literacy	Health literacy (HL) 5
Perceived organizational support	Perceived organizational support (POS) by Eisenberger R.
Personality	Big 5
*Outcomes*	
Psychological distress	Kessler 6 (K6)
Work engagement	Utrecht Work Engagement Scale (UWES-9)
Self-rated health	Direct inquiry questions
Presenteeism	World Health Organization Health and Work Performance Questionnaire (HPQ)
Sickness presenteeism	Direct inquiry questions
Turnover intention	Direct inquiry questions
Sense of belonging to company	Employee’s net promoter score
*Workplace accident related*	
Near-miss experience	Direct inquiry questions
Occupational accident	Direct inquiry questions
Slip, fall, and trip	Direct inquiry questions

### Statistical analysis

We have indicated the number of participants for analysis and those we judged to have given invalid responses by sex and age category. We compared the differences between those two groups using *t* tests for continuous variables (such as age, K6, and work engagement score) and chi-square tests for categorical variables (such as sex and nationality). We determined age, gender, nationality, marital status, household annual income, employment status, industry, K6, and work engagement score by sex for the participants who excluded invalid responses.

## Results

We were able to obtain the desired number of participants for sex, age, and regional stratification. Detailed information based on the CHERRIES appears in Table 3. We have no information of the view rate and participants rate owing to the confidentiality policy of the survey company. We have also no information of the completion rate, while 59,272 registered monitors answered the initial screening questions and participated in the survey; of those, 29,997 matched the survey’s criteria (regarding worker status, region, sex, and age). Thus, the completion rate is greater than 50.6% (29,997/59,272).

**Table 3:** Checklist for reporting results of internet E-Surveys (CHERRIES).

Item category	Checklist item	Explanation	Response
*Design*			
	Describe survey design	Describe target population, sample frame. Is the sample a convenience sample? (In open surveys, this is most likely.)	The survey is commissioned by Rakuten Insight, Inc. (Tokyo, Japan), which has 2.2 million registered monitors. The company sends participation information to registered monitors, so this is not an open survey.
*Institutional review board (IRB) approval and informed consent*			
	IRB approval	Whether the study is approved by an IRB	Yes The study is approved by the Ethics Committee of the University of Occupational and Environmental Health, Japan (R3-076).
	Informed consent	Informed consent process: are the participants told the duration of the survey, which data are stored, where, and for how long, who the investigator is and the study purpose?	Only those who give their consent complete the questionnaire. A document describing the duration of the survey, which data are stored, where, and for how long, who the investigators is, and the study purpose is available on the department website of one of the researchers (https://www.ohpm.jp/information/ (in Japanese).
	Data protection	If any personal information is collected or stored, what mechanisms are used to protect unauthorized access?	We do not collect personal information. Respondents are contacted in accordance with the privacy policy of Rakuten Insight
*Development and pretesting*			
	Development and testing	How the survey was developed, including whether the usability and technical functionality of the electronic questionnaire were tested before fielding the questionnaire	Several researchers answer Web questions. They check inappropriate expressions, ease of answering and other issues, and revise the content.
*Recruitment process and participants having access to the questionnaire*			
	Open versus closed survey	An open survey is accessible to each visitor to a site; a closed survey is accessible only to a sample identified by the investigator (password-protected survey).	Only people registered with Rakuten Insight can respond to the survey.
	Contact mode	Whether or not the initial contact with potential participants is made on the Internet. (Investigators may also send out questionnaires by email and allow for Web-based data entry.)	Available only on the Internet
	Advertising the survey	How and where the survey is announced or advertised: some examples are offline (newspapers) and online media (mailing lists—if yes, which?) and banner ads (where the banner ads are posted and their appearance).The wording of the announcement is important: it heavily influences who chooses to participate. Ideally, the survey announcement should be published as an Appendix.	Only people registered with Rakuten Insight can answer this Internet survey. The company sends participation information to registered monitors, explaining they will receive a certain number of bonus points if they respond.
*Survey administration*			
	Web or e-mail	Type of e-survey (e.g., posted on a web site or sent via e-mail). If an e-mail survey, are the responses entered manually into a database or is there an automatic method for receiving responses?	Rakuten Insight sends documentation via e-mail. There is an automatic method for receiving responses.
	Context	Type of website (for mailing lists or newsgroups) in which the survey is posted: what is the website about, who visits it, and what do visitors normally seek? To what degree does the website content preselect the sample or influence results. For example, a survey about vaccination on an anti-immunization site would yield different results from a survey conducted on a government website	Visitors preregister as survey members. Recruitment channels are various Rakuten Group sites and some external media (https://member.insight.rakuten.co.jp/). The visitors’ purpose is to answer the survey and accumulate bonus points; there is no content other than the survey material on the page after logging in; thus, the impact on results is considered small.
	Mandatory or voluntary	Is it a mandatory survey to be completed by every visitor who wished to access the website, or is it a voluntary survey?	It is a voluntary survey.
	Incentives	Were any incentives offered (e.g., monetary, prizes, or non-monetary incentives, such as an offer to provide the survey results)?	Yes. Survey respondents can earn redeemable points as an incentive from the survey company; however, owing to company confidentiality, the researchers are unaware of the number of points.
	Study period	Data-collection period	Feburary 28 to March 3, 2022
	Randomization of items or questionnaires	To prevent biases, whether survey items could be randomized or alternated	No
	Adaptive questioning	Use of adaptive questioning (with certain questionnaire items, or only conditionally displayed based on responses to other items) to reduce question number and complexity	Yes
	Number of items	Number of questionnaire items per page: the number is an important factor for the completion rate.	One item per page
	Number of screens (pages)	Number of pages over which questionnaire distributed: the number is an important factor for the completion rate.	77 pages
	Completeness check	It is technically possible to do consistency or completeness checks before questionnaire submission. Is this done? If yes, how (usually JAVAScript)? An alternative is to check for completeness after questionnaire submission (and highlight mandatory items). If it was done, it should be reported. All items should provide a nonresponse option (such as “inapplicable” and “rather not say”), and selection of one response option should be enforced.	We do consistency or completeness checks before submission. With inconsistency in a question, an alert is displayed; for example, participants who list meaningless characters and symbols in the free-answer column (e.g., “AAA” and “$&¥”) and those who respond in an impossibly short time.
	Review step		Yes
*Response rates*			
	Unique site visitor	If you provide view or participation rates, you need to define how you determine a unique visitor. There are different techniques available, based on IP addresses or cookies or both.	We use the monitor ID given to respondents when they access the survey system.
	View rate (ratio of unique survey visitors to unique site visitors)	This requires counting unique visitors to the first page of the survey divided by the number of unique site visitors (not page views!). It is not unusual to have view rates of under 0.1% with a voluntary survey.	No information is available on the number of invitations to participate owing to the confidentiality policy of the monitor company.
	Participation rate (ratio of unique visitors who agreed to participate to unique first-survey page visitors)	Count the unique number of people who fill in the first-survey page (or agree to participate, e.g., by marking a checkbox) divided by visitors who visit the first page of the survey (or the informed-consent page, if present). This can also be termed “recruitment rate.”	No information is available owing to the confidentiality policy of the monitor company; 59,272 registered monitors answered the initial screening questions.
	Completion rate (ratio of users who finished the survey to users who agreed to participate)	The number of people submitting the last questionnaire page divided by the number of those who agree to participate (or submit the first-survey page). This is only relevant if there is a separate informed-consent page or if the survey extends over several pages. This is a measure for attrition. Note that “completion” can involve leaving questionnaire items blank. This is not a measure for how completely questionnaires are filled in. (If you a measure for this is needed, use the expression “completeness rate.”)	No information is available on the number of invitations to participate owing to the confidentiality policy of the monitor company; 59,272 registered monitors answered the initial screening questions and participated in the survey; of those, 29,997 matched the survey’s criteria (regarding worker status, region, sex, and age). Thus, the completion rate is greater than 50.6% (29,997/59,272).
*Preventing multiple entries from the same individual*			
	Cookies used	Whether cookies are used to assign a unique user identifier to each client computer: if so, indicate the page on which the cookie is set and read, and how long the cookie is valid. Are duplicate entries avoided by preventing user access to the survey twice? Or are duplicate database entries having the same user ID eliminated before analysis? If the latter, which entries are kept for analysis (e.g., first entry or most recent)?	No
	IP check	Whether the IP address of the client computer is used to identify potential duplicate entries from the same user: if so, indicate the period over which no two entries from the same IP address are allowed (e.g., 24 h). Are duplicate entries avoided by preventing users with the same IP address accessing the survey twice? Or are duplicate database entries having the same IP address within a given period eliminated before analysis? If the latter, which entries are kept for analysis (e.g., first entry or most recent)?	No
	Log file analysis	Whether other techniques to analyze the log file for identification of multiple entries are used: if so, describe	No
	Registration	In closed (non-open) surveys, users need to log in first, and it is easier to prevent duplicate entries from the same user. Describe how this is done. For example, is the survey never displayed a second time once the user completes it, or is the user name stored together with the survey results and later eliminated? If the latter, which entries are kept for analysis (e.g., first entry or most recent)?	It is managed by the member ID.
*Analysis*			
	Handling of incomplete questionnaires	Whether only completed questionnaires are analyzed: are questionnaires that terminated early also analyzed (e.g., when users do not complete all questionnaire pages)?	We analyze only completed questionnaires.
	Questionnaires submitted with an atypical time stamp	Some investigators may measure the time people needed to complete a questionnaire and exclude questionnaires that are submitted too early. Specify the time frame that is used as a cutoff point, and describe how that point was determined.	We exclude responses that are answered excessively early in the survey. However, researchers are unable to ascertain the time taken to complete questionnaires because that information is subject to survey company confidentiality.
	Statistical correction	Whether methods such as weighting of items or propensity scores are used to adjust for non-representative samples: if so, indicate the methods	No

IP address: Internet protocol address.

Table 4 presents specifics of the valid and invalid participants. The valid respondents were 27,693 and the invalid respondents were 2,304. The invalid responses comprised 1,244 (7.6%) men and 1,060 (7.8%) women. The proportion of invalid respondents ranged from 6.4% of women in their 30s to 11.2% of women aged over 65 years.

**Table 4:** Valid and invalid respondents stratified by sex and age category.

Age category (years)	Valid respondents (*n* = 27,693)		Invalid respondents (*n* = 2,304)	
	Men (15,201)	Women (12,492)	Men (1,244)	Women (1,060)
20–29	2,366	2,188	237 (9.1%)	191 (8.0%)
30–39	2,896	2,355	246 (7.8%)	160 (6.4%)
40–44	1,722	1,444	152 (8.1%)	116 (7.4%)
45–49	1,999	1,715	141 (6.6%)	138 (7.4%)
50–54	1,731	1,489	129 (6.9%)	111 (6.9%)
55–59	1,506	1,235	115 (7.1%)	112 (8.3%)
60–64	1,238	922	93 (7.0%)	87 (8.6%)
≥65	1,743	1,144	131 (7.0%)	145 (11.2%)

Table 5 shows the demographic characteristics and mental health–related indicators (K6 and work engagement score) for the valid and invalid participants. No differences were found between the groups for age, sex, and nationality. However, the invalid participants had statistically significantly higher psychological distress (*p* < 0.001) and marginal significantly lower work engagement (*p* = 0.075) than the valid participants.

**Table 5:** Sociodemographic and mental health-related factors among valid and invalid respondents.

	Valid respondents (27,693)	Invalid respondents (2,304)	*p* value
Age, mean (SD)	45.8 (13.5)	45.9 (14.3)	0.530
Sex (men), n (%)	15,201 (54.9%)	1,244 (54.0%)	0.410
Nationality (Japan), *n* (%)	27,524 (99.4%)	2,289 (99.3%)	0.810
K6, mean (SD)	4.3 (4.8)	4.7 (5.3)	<0.001
WE, mean (SD)	2.5 (1.4)	2.4 (1.4)	0.075

SD, standard deviation; K6, Kessler 6; WE, work engagement.

Table 6 summarizes the characteristics of the valid participants by sex. The number and mean age of men were 15,201 (55%) and 46 years; those of women were 12,492 (45%) and 45 years. We observed that 0.6% of valid participants had differences between their biological and self-perceived sex. In all, 59% of men were married compared with 51% for women. Regarding employment status, 66% of men were permanent employees; 41% of women were permanent employees, and 34% of women had part-time employment. By industry, 21% of men and 10% of women worked in manufacturing; 8% of men and 21% of women worked in medical, health care and welfare; and 8% of men and 4% of women worked in the public sector. Regarding mental health status, 35% of men and 40% of women had a K6 score of 5 or higher, indicating mild psychological distress.

**Table 6:** Valid participant characteristics by sex.

		Total	Sex	
		27,693	Men (15,201)	Women (12,492)
Age, mean (SD)		45.8 (13.5)	46.3 (13.6)	45.1 (13.4)
*Gender, n (%)*				
Male	15,159 (54.7%)	15,120 (99.5%)	39 (0.3%)	Female	12,437 (44.9%)	43 (0.3%)	12,394 (99.2%)	Other	97 (0.4%)	38 (0.2%)	59 (0.5%)
	Nationality (Japanese), n (%)		27,524 (99.4%)	15,105 (99.4%)	12,419 (99.4%)
	*Marital status, n (%)*				
Married	15,241 (55.0%)	8,928 (58.7%)	6,313 (50.5%)	Unmarried	8,362 (30.2%)	4,497 (29.6%)	3,865 (30.9%)	Divorced or bereaved	4,090 (14.8%)	1,776 (11.7%)	2,314 (18.5%)
	*Household income (yen), n (%)*				
	<4 million	6,926 (25.0%)	3,100 (20.4%)	3,826 (30.6%)	4 million–6 million	6,919 (25.0%)	3,918 (25.8%)	3,001 (24.0%)	6 million–8 million	5,478 (19.8%)	3,138 (20.6%)	2,340 (18.7%)	8 million–10 million	3,814 (13.8%)	2,232 (14.7%)	1,582 (12.7%)	10 million–12 million	1,871 (6.8%)	1,163 (7.7%)	708 (5.7%)	>12 million	2,685 (9.7%)	1,650 (10.9%)	1,035 (8.3%)
*Employment status, n (%)*				
Self-employed		2,596 (9.4%)	1,654 (10.9%)	942 (7.5%)	Company executive	1,595 (5.8%)	1,213 (8.0%)	382 (3.1%)	Full-time employee	15,240 (55.0%)	10,064 (66.2%)	5,176 (41.4%)	Part-time work	5,217 (18.8%)	936 (6.2%)	4,281 (34.3%)	Dispatched employee	574 (2.1%)	158 (1.0%)	416 (3.3%)	Contract employees	1,747 (6.3%)	888 (5.8%)	859 (6.9%)	Freelance	207 (0.7%)	33 (0.2%)	174 (1.4%)	Other	517 (1.9%)	255 (1.7%)	262 (2.1%)
		*Industry category, n (%)*				
		Agriculture and forestry	261 (0.9%)	172 (1.1%)	89 (0.7%)	Fisheries	22 (0.1%)	13 (0.1%)	9 (0.1%)	Mining and quarrying of stone and gravel	29 (0.1%)	23 (0.2%)	6 (<0.1%)	Construction	1,399 (5.1%)	980 (6.4%)	419 (3.4%)	Manufacturing	4,432 (16.0%)	3,246 (21.4%)	1,186 (9.5%)	Electricity, gas, heat supply, and water	391 (1.4%)	295 (1.9%)	96 (0.8%)	Information and communications	1,369 (4.9%)	982 (6.5%)	387 (3.1%)	Transport and postal services	1,221 (4.4%)	909 (6.0%)	312 (2.5%)	Wholesale and retail trade	2,887 (10.4%)	1,292 (8.5%)	1,595 (12.8%)	Finance and insurance	1,197 (4.3%)	549 (3.6%)	648 (5.2%)	Real estate and goods rental and leasing	702 (2.5%)	452 (3.0%)	250 (2.0%)	Scientific research, professional, and technical services	788 (2.8%)	494 (3.2%)	294 (2.4%)	Accommodations, eating, and drinking services	968 (3.5%)	326 (2.1%)	642 (5.1%)	Living-related and personal services and amusement services	749 (2.7%)	294 (1.9%)	455 (3.6%)	Education and learning support	1,718 (6.2%)	719 (4.7%)	999 (8.0%)	Medical, health care and welfare	3,775 (13.6%)	1,218 (8.0%)	2,557 (20.5%)	Compound services	267 (1.0%)	154 (1.0%)	113 (0.9%)	Services	2,835 (10.2%)	1,469 (9.7%)	1,366 (10.9%)	Public sector	1,645 (5.9%)	1,165 (7.7%)	480 (3.8%)	Unlabeled	1,038 (3.7%)	449 (3.0%)	589 (4.7%)
			K6, mean (SD)		4.3 (4.8)	4.0 (4.7)	4.6 (4.9)
	K6 (5 and more), *n* (%)		10,328 (37.3%)	5,285 (34.8%)	5,043 (40.4%)
	K6 (10 and more), *n* (%)		3,967 (14.3%)	2,023 (13.3%)	1,944 (15.6%)
	K6 (13 and more), *n* (%)		1,916 (6.9%)	952 (6.3%)	964 (7.7%)
	WE, mean (SD)		2.5 (1.4)	2.4 (1.4)	2.5 (1.4)

K6, Kessler 6; WE, work engagement; SD, standard deviation.

## Discussion

We conducted an Internet-based survey of workers in Japan in March 2022. We reported the details of the study based on the CHERRIES. Our sample had the same sex, age, and regional composition as the Japanese working population.

When a rapid change occurs in society or the environment such as a pandemic, it is necessary to conduct a rapid survey to understand the situation. We conducted a survey via the Internet on a panel registered with a research firm. As a result, we were able to gather a large sample of approximately 30,000 people in a short period of four days. This indicates that web-based surveys can be a powerful tool for conducting surveys quickly. However, it is necessary to provide details regarding the recruitment process and description of the sample. This protocol paper is intended to disclose this information.

CHERRIES requires disclosure of the recruitment process. This study included monitors registered with Rakuten Insight, Inc. The Rakuten Group has over 100 million individuals in Japan at the time of the survey who hold IDs related to Rakuten’s services (18). Even taking into account that one person can afford multiple IDs, many people in Japan use Rakuten’s services, and for the purpose of sampling as a standard population for Japan as a whole, we believed that the bias was not significant. Furthermore, the strength of this study is that we stratified the sampling by sex, age, and geographic area to ensure that the sample is representative of the standard population of workers in Japan.

It is also important to identify the analysis population by excluding inappropriate or incomplete responses. We took steps to exclude participants who made dishonest responses. Among the invalid respondents, we found no bias regarding sex, age, or nationality. We conducted our survey only in Japanese language and foreign nationals proficient in that language. Invalid participants exhibited higher levels of psychological distress and lower work engagement than the valid ones. Individuals with poor mental health are subject to greater fatigue and have lower attention spans, which increase the likelihood of invalid responses (19). Alternatively, giving inaccurate answers may be associated with psychological distress and poor work engagement (20).

Among our valid participants, 0.6% indicated differences with respect to sex and gender. In one Japanese survey conducted in 2019 (21), 0.7% of respondents answered no to the question “Do you see your gender now as the same as your sex at birth?” That survey was conducted on a general population (of whom 80% were workers); however, the proportion of individuals with differences regarding sex and gender was almost the same as in our survey. We found that non-Japanese participants accounted for 0.6% of the total. According to a nationwide survey, the number of foreign workers in Japan was 1,727,221 as of October 31, 2021, and they accounted for 2.5% of the overall workforce (22). The low percentage of non-Japanese participants in our survey suggests that few of them were registered with that Internet panel. The reason is that surveys conducted by research firms are usually conducted in Japanese, so it is highly likely that non-Japanese with limited Japanese reading skills are not registered in the Internet panel. It may be inappropriate to target panels registered with Internet research firms in order to conduct surveys on foreign workers. It also suggests that the survey should be conducted taking account the diversity of languages used.

We found that the proportion of married participants was 58.7% for men and 50.5% for women. According to the national census of 2020 (23), 57.4% of married people in Japan were men and 54.0% were women; in 2015, the figures were 58.9 and 55.2%, respectively; thus, a decrease occurred for both sexes. We found that among our valid participants, the proportion of married people was higher for men and lower for women than in the general population (7). When asked if obstacles existed to getting married within the next year, one study found that over 40% of both men and women cited wedding costs and funding necessary to set up a new life (24). Financial reasons may deter unemployed men from getting married. By contrast, women may defer marriage through having to stop work following marriage or childbirth. According to one study, the average household income in Japan in 2020 was 5,643,000 yen (25). We found that 53.8% of men and 45.4% of women had annual household incomes of 6 million yen or more; thus, our participants had similar income levels to the overall worker population in Japan.

Regarding employment status, our participants displayed similar proportions to those identified in a nationwide statistical survey in Japan (25), indicating that our sampling was appropriate. In the national survey, 11.4% of men and 8.6% of women were self-employed; in our study, those figures were 10.9 and 7.5%, respectively. Among workers excluding self-employed individuals and company executives, the proportion of full-time employees was 77.8% for men and 46.6% for women in that national survey; in our study, those figures were 81.6 and 46.3%, respectively. Similar findings emerged with respect to industry. Manufacturing accounts for the largest number of employees: the national survey found that 20.5% of men and 10.6% of women worked in that sector; our results indicated 21.4% of men and 9.5% of women, respectively. Our study was conducted approximately 2 years after COVID-19 began. We found that medical, health care and welfare accounted for 8.0% of men and 20.5% of women; those figures do not markedly differ from the 6.4 and 23.1%, respectively, in the national survey. Thus, we believe that COVID-19 did not prevent health workers from participating in our study.

We used the K6 score for depression and anxiety to assess mental health status. A K6 score of 10 or higher indicates experiencing psychological distress equivalent to mood or anxiety disorders (26). The proportion of our participants with a score of 10 or higher (13.3% for men and 15.6% for women) was exactly the same as observed in the national survey (26). Conversely, the proportions of participants scoring 5 or higher (moderate risk of depression and anxiety) were 29.6% for men and 34.8% for women in the national survey in 2019; we recorded 34.8 and 40.4%, respectively. That national survey was based on data from before COVID-19. Two years after the pandemic began, the levels of depressive and anxiety disorders were not so high; however, many people still apparently experienced mild anxiety. Work engagement is a positive aspect of mental health; it was found to be 2.4 for men and 2.4 for women in a large Japanese survey conducted after the start of the pandemic (27); in this study we recorded figures of 2.4 and 2.5, respectively.

In conclusion, the present study describes in detail the protocol of a baseline survey for a cohort study of the Japanese workforce population. We stratified sex, age, and region such that our population ratios matched the nationwide situation. We also reported the details of the study based on the CHERRIES. Our results indicate that the sampling was adequate in terms of employment status and industry. We plan to use this database to develop benchmarks related to occupational health and safety.

## Ethics statement

The study involving humans were approved by the Ethics Committee of the University of Occupational and Environmental Health, Japan (R3-076). The studies were conducted in accordance with the local legislation and institutional requirements. The ethics committee/institutional review board waived the requirement of written informed consent for participation from the participants or the participants’ legal guardians/next of kin because the survey was conducted online, therefore no written consent was obtained. However, an explanatory document was presented and a check box was provided to obtain confirmation of consent. Consent was obtained from all respondents.

## Author contributions

TN, KO, and KM, study design. TN, KO, NA, MN, and KM, data collection. TN data analysis and draft of manuscript. All authors have reviewed, edited and approved the final manuscript.
