# Supplementary material for: Protocol of a study to benchmark occupational health and safety in Japan: W2S-Ohpm study
Source: Front Public Health. 2023 Nov 3;11:1191882. doi: 10.3389/fpubh.2023.1191882 (PMC10655078; doi:10.3389/fpubh.2023.1191882)
Supplement: Supplementary file 1 [file Table_1.pdf]

Supplementary Material

Table 1

	W2S-Ohpm			The national survey		
	Total	Sex		Total	Sex	
		Men	Women		Men	Women
	(n, %)	(n, %)	(n, %)	(n (million), %)	(n (million), %)	(n (million), %)
Total	27,693	15,201	12,492	6,687	3,678	3,011
Self-employed	2,596 (9.4%)	1,654 (10.9%)	942 (7.5%)	647 (9.7%)	402 (10.9%)	245 (8.1%)
Company executive	1,595 (5.8%)	1,213 (8.0%)	382 (3.1%)	342 (5.1%)	259 (7%)	83 (2.8%)
Full-time employee	15,240 (55.0%)	10,064 (66.2%)	5,176 (41.4%)	3,597 (53.8%)	2,348 (63.8%)	1,250 (41.5%)
Part-time work	5,217 (18.8%)	936 (6.2%)	4,281 (34.3%)	1,474 (22%)	349 (9.5%)	1,126 (37.4%)
Dispatched employee	574 (2.1%)	158 (1.0%)	416 (3.3%)	149 (2.2%)	59 (1.6%)	90 (3%)
Contract employees	1,747 (6.3%)	888 (5.8%)	859 (6.9%)	395 (5.9%)	221 (6%)	174 (5.8%)
Freelance/Other	724 (2.6%)	288 (1.9%)	436 (3.5%)	83 (1.2%)	40 (1.1%)	43 (1.4%)
Total	27,693	15,201	12,492	6,514	3,574	2,940
Agriculture and forestry	261 (0.9%)	172 (1.1%)	89 (0.7%)	192 (2.9%)	118 (3.3%)	74 (2.5%)
Construction	1,399 (5.1%)	980 (6.4%)	419 (3.4%)	479 (7.4%)	394 (11%)	85 (2.9%)
Manufacturing	4,432 (16.0%)	3,246 (21.4%)	1,186 (9.5%)	1044 (16%)	732 (20.5%)	312 (10.6%)
Information and communications	1,369 (4.9%)	982 (6.5%)	387 (3.1%)	272 (4.2%)	195 (5.5%)	77 (2.6%)
Transport and postal services	1,221 (4.4%)	909 (6.0%)	312 (2.5%)	351 (5.4%)	274 (7.7%)	77 (2.6%)

Wholesale and retail trade	2,887 (10.4%)	1,292 (8.5%)	1,595 (12.8%)	1044 (16%)	497 (13.9%)	547 (18.6%)
Finance and insurance	1,197 (4.3%)	549 (3.6%)	648 (5.2%)	160 (2.5%)	73 (2%)	87 (3%)
Real estate and goods rental and leasing	702 (2.5%)	452 (3.0%)	250 (2.0%)	141 (2.2%)	83 (2.3%)	58 (2%)
Scientific research, professional and technical services	788 (2.8%)	494 (3.2%)	294 (2.4%)	254 (3.9%)	158 (4.4%)	96 (3.3%)
Accommodations, eating and drinking services	968 (3.5%)	326 (2.1%)	642 (5.1%)	381 (5.8%)	145 (4.1%)	236 (8%)
Living-related and personal services and amusement services	749 (2.7%)	294 (1.9%)	455 (3.6%)	225 (3.5%)	89 (2.5%)	136 (4.6%)
Education and learning support	1,718 (6.2%)	719 (4.7%)	999 (8.0%)	349 (5.4%)	145 (4.1%)	204 (6.9%)
Medical, health care and welfare	3,775 (13.6%)	1,218 (8.0%)	2,557 (20.5%)	908 (13.9%)	227 (6.4%)	680 (23.1%)
Compound services/Services	3,102 (11.2%)	1,623 (10.7%)	1,479 (11.8%)	463 (7.1%)	273 (7.6%)	190 (6.5%)
Public sector	1,645 (5.9%)	1,165 (7.7%)	480 (3.8%)	251 (3.9%)	171 (4.8%)	81 (2.8%)
Fisheries	22 (0.1%)	13 (0.1%)	9 (0.1%)			
Mining and quarrying of stone and gravel	29 (0.1%)	23 (0.2%)	6 (<0.1%)			
Electricity, gas, heat supply and water	391 (1.4%)	295 (1.9%)	96 (0.8%)			
Unlabeled	1,038 (3.7%)	449 (3.0%)	589 (4.7%)			
